# The Regulatory Role of MicroRNAs on Phagocytes: A Potential Therapeutic Target for Chronic Diseases

**DOI:** 10.3389/fimmu.2022.901166

**Published:** 2022-05-11

**Authors:** Yongbo Wang, Xingyu Liu, Panpan Xia, Zhangwang Li, Xinxi FuChen, Yunfeng Shen, Peng Yu, Jing Zhang

**Affiliations:** ^1^ Department of Metabolism and Endocrinology, The Second Affiliated Hospital of Nanchang University, Jiangxi, China; ^2^ The Second Clinical Medical College of Nanchang University, The Second Affiliated Hospital of Nanchang University, Jiangxi, China; ^3^ Department of Anesthesiology, The Second Affiliated Hospital of Nanchang University, Jiangxi, China

**Keywords:** chronic disease, miRNA, immunity, neutrophils, macrophages

## Abstract

An effective acute inflammatory response results in the elimination of infectious microorganisms, followed by a smooth transition to resolution and repair. During the inflammatory response, neutrophils play a crucial role in antimicrobial defense as the first cells to reach the site of infection damage. However, if the neutrophils that have performed the bactericidal effect are not removed in time, the inflammatory response will not be able to subside. Anti-inflammatory macrophages are the main scavengers of neutrophils and can promote inflammation towards resolution. MicroRNAs (miRNAs) have great potential as clinical targeted therapy and have attracted much attention in recent years. This paper summarizes the involvement of miRNAs in the process of chronic diseases such as atherosclerosis, rheumatoid arthritis and systemic lupus erythematosus by regulating lipid metabolism, cytokine secretion, inflammatory factor synthesis and tissue repair in two types of cells. This will provide a certain reference for miRNA-targeted treatment of chronic diseases.

## 1 Introduction

Inflammation is a cascade reaction of human tissues and organs response to harmful stimuli such as pathogens (1). On the cellular level, inflammation that is manifested as mutual damage between damage factors and histiocytes, as well as the regeneration of parenchymal cells and interstitial cells are often followed by tissue dysfunction due to changes in protein activity, changes in cellular metabolites, and connective tissue reorganization (2). The solution of inflammation mainly includes two aspects: one is anti-inflammatory, that is to prevent the re-recruitment of inflammatory cells; the other is decomposition, that is to remove apoptotic inflammatory cells (mainly neutrophils) (3). However, unresolved inflammatory cascades that bring new features to tissues and cells may promote he establishment of chronic inflammation leading to tissue and organ dysfunction (4). Thus, chronic inflammation can contribute to many potential chronic diseases, including diabetes (5), cardiovascular disease (CVD) (6), rheumatoid arthritis (RA) (7), inflammatory bowel disease (IBD) (8), neurodegenerative diseases (9) and systemic lupus erythematosus (SLE) (10).

Both neutrophils and macrophages belong to phagocytic cells, but they play different important roles in inflammatory response. Neutrophils are “whistlers” of the inflammatory response, which means they are the first immune cells to be recruited to the site of infection or injury. The phagocytosis of neutrophils can destroy pathogens and some damage factors. However, if it is not cleared in time, the derived death induction pathways such as oxidation and hydrolysis can form an inflammation amplification loop, causing serious tissue damage and developing the inflammatory response into a chronic disease (11). Inflammatory cells such as neutrophils are often eliminated by macrophages. Macrophages can not only phagocytose pathogens and damage factors, but also phagocytose apoptotic cells and participate in lipid metabolism. For example, the efferocytosis of macrophages enables the apoptotic cells to be eliminated before necrosis, releasing anti-inflammatory cytokines and specialized proresolving mediators (SPMs) at the same time, and establishing immune tolerance (12). Therefore, macrophages are the “finalizers” of the inflammatory response and play a key role in the resolution and regression of inflammation. Macrophages play distinct roles in the different stages of inflammatory response. The dysfunction of macrophages in the late stage of the inflammatory response is likely to prevent the inflammation from resolving.

Studies on the relationship between non-coding RNAs (ncRNA) and the control of chronic inflammatory diseases in some species have shown that ncRNA has become a key regulatory factor for the development and function of the immune system (13–15). miRNAs belong to a major subfamily of ncRNA and are endogenous non-coding ribonucleic acid with a length of about 20 nucleotides (16). miRNAs mediate specific gene silencing through complementarity to mRNA sequences (17, 18). A miRNA can be targeted to multiple mRNAs, while an mRNA can also be targeted by many different or related miRNAs. miRNAs often control multiple targets within a signal axis to amplify and regulate the utility. In addition to directly targeting mRNAs, miRNAs can further coordinate inflammation by targeting enzymes or transcription factors exerting indirect effects (16, 18–21). These basic properties of miRNA make it very suitable for regulating chronic inflammatory (2). miRNAs regulate inflammatory cascades by regulating target gene levels, which determine whether phagocytosis occurs, how strong the response is, and the threshold at which inflammation subside (16). For example, under the stimulation of inflammatory mediators and mildly oxidized low-density lipoprotein, the expression of miR-155 is up-regulated. MiR-155 directly targets B-cell lymphoma 6 protein (Bcl6), enhances the expression of inflammatory mediators (such as CCL2) in macrophages, and damages the efferent cytosis of macrophages, leading to the accumulation of inflammatory cells at the infected site of injury and secondary necrosis of apoptotic cells, which eventually develops into atherosclerosis (22).

The existing literature on miRNA is extensive and focuses particularly on the regulation of various signaling pathways by miRNA. In recent years, there has been growing recognition of the vital links between miRNAs and immune cells. miRNAs are active in inflammatory responses. For example, miRNAs regulate the levels and types of chemokines by targeting chemokine (C-X-C motif) ligand (CXCL) (23), cholesterol metabolism by targeting ATP binding cassette transporter A1 (ABCA1) and ATP binding cassette transporter G1 (ABCG1) (24), the release of inflammatory factors by targeting the nuclear factor kappa-B (NF-κB) signaling pathway (25), and tissue repair by targeting suppressor of cytokine signaling-1 (SOCS1) (26).

Although there are many reports in the literature on regulation of specific miRNA on phagocytes (neutrophils and macrophages), most are restricted to functional elucidations without reference to specific chronic diseases. There has been very little systematic summery of the regulations of miRNA on phagocytes in specific diseases. We fills a gap in the research on this aspect. The primary aim of this paper is to review recent research into the regulation mechanism of miRNA on phagocytes in different chronic diseases, explore the relationship between miRNAs and phagocytes in different chronic diseases, and provide empirical evidence for the claim that miRNA can become a potential therapeutic target for chronic diseases eventually.

## 2 Mechanism of Inflammatory Response and Chronic Inflammation

### 2.1 Function and Mechanism of Inflammatory Response

Under normal conditions, the homeostatic control mechanism maintains the acceptable range of the environmental parameters near the predetermined equilibrium point (27). Abnormal conditions may cause certain parameters to deviate from their normal homeostasis range, resulting in stress response (28). Acute and chronic inflammation are two distinct adaptive stresses triggered by inadequate or ineffective other homeostatic mechanisms. When body tissue is damaged, the inflammatory process is a protective cascade of local blood vessels characterized by redness, swelling, pain, heat and dysfunction, which can aid in the removal of foreign bodies and tissue repair (29, 30). The inflammatory cascade is preprogrammed and pervasive, and can be triggered by almost every tissue, playing an important physiological role in tissue homeostasis (31).

When the body faces threats such as infection and injury, it induces its own acute inflammatory response, which usually lasts for minutes to days (32). The inflammatory response is controlled by a complex regulatory networds consisting of inducers, sensors, mediators, and effectors, and the inflammatory response can be determined according to the components of the regulatory networks (33). The recognition of pattern recognition receptors (PRR) initiates an immune response that identifies structural components of pathogens as pathogen-associated molecular patterns (PAMPs) and chemicals produced by injured cells as damage-associated molecular patterns (DAMPs) (34–36). Different receptors in immune cells recognize these patterns, and upon triggering these receptors, inflammatory cytokines such as TNF-α and IL-6 are released, causing changes in endothelial cells and allowing immune cells to flow through endothelial cells to tissues (29, 37). DAMPs are a class of substances released by cells into the surrounding interstitial fluid when cells are stimulated by injury, hypoxia and stress, including degraded matrix molecules, leukocyte degranulation molecules, and heat shock proteins (HSP). They are considered harmful signals that trigger inflammatory responses through pattern recognition receptors such as Toll-like receptors or NOD (nucleotide binding oligomer domain protein)-like receptors (34–36, 38). For example, TLR4 can identify endogenous substrates such as free fatty acids (FFA) (38). PAMPs are ligand receptors that are recognized and bound by PRRs, including some highly conserved molecular structures shared on the surface of pathogenic microorganisms, such as the lipopolysaccharide of PAMPs are ligand receptors that are recognized and bound by PRRs, including some highly conserved molecular structures shared on the surface of pathogenic microorganisms, such as the lipopolysaccharide of G^-^ (gram-negative) bacterium. They also include some common molecular structures on the surface of host apoptotic cells, such as phosphatidyl serine (33). NADPH oxidase utilizes the respiratory burst in hexose phosphate to convert oxygen molecules into superoxide anions. Superoxide anion generates hydrogen peroxide under the action of superoxide dismutase (SOD), which can be bactericidal, and can further generate oxides with strong bactericidal power under the action of myeloperoxide (MPO), such as OCI^-^. However, reactive oxygen species (ROS) generated during oxidative sterilization of neutrophils may leak into surrounding tissues. Strong bactericidal oxidants may also cause damage to neutral proteases that inhibit lysosome release, thereby harming surrounding tissues (39).

Infection and tissue damage are well-known inflammatory triggers that attract leukocytes and plasma proteins to tissue damage (4). Furthermore, tissue stress or dysfunction induces an adaptive response called para-inflammation, which occurs between essential homeostasis and canonical inflammatory responses and is mediated primarily by tissue-resident macrophages (4, 40). Regardless of the cause of the inflammatory response, the ultimate goal is to eliminate or isolate the source of the interference, allowing the host to adapt to the abnormal condition and restore tissue function and homeostasis. However, part of the inflammation can develop into chronic disease due to persistent tissue dysfunction due to environmental variables, genetic mutations and even modern unhealthy human diseases (31, 41).

### 2.2 The Role of Neutrophils and Macrophages in the Inflammatory Response

At the most basic level, acute inflammatory responses induced by infection or tissue damage result in the coordinated distribution of blood components (plasma and leukocytes) to the site of disease or injury. This process can be roughly divided into: First, blood vessel flow and tiny blood vessels become larger, and newly formed capillaries and larger arterioles help increase blood flow to areas of inflammation (4). Then, vasodilation and vascular permeability increase, leading to leakage of microcirculatory plasma and phagocytosis of leukocytes. Endothelial cell selectins on the surface of vascular endothelial cells can inducibly connect with leukocyte integrins and chemokine receptors. This connection allows endothelial cell modification, increased microvascular permeability, and preferential access of plasma proteins and white blood cells (mainly neutrophils) to infected or injured tissues through the posterior capillary vein (32). Neutrophils are activated by direct stimulation of cytokines released by pathogens or tissue resident cells, and activated neutrophils capture bacteria *via* phagosomes and then begin almost simultaneously degranulation-dependent non-oxygen bactericidal action and triphosphopyridine nucleotide (NADPH) oxidation enzyme (NOX2)-dependent aerobic bactericidal action (42). Under the degranulation-dependent non-oxygen sterilization, various bactericides and hydrolytic enzymes in the granules are released, including lysozyme, bactericidal/permeability increasing protein polypeptide (BPI) protein, defensin, elastase, cathepsin G, protease 3, azurocidin (CAP37) and acid β-glycerophosphatase (43). However, if these bactericides and hydrolases in granules are exocytosed into tissues outside neutrophils, they will damage normal tissues and further aggravate inflammation (44). In oxygen-dependent sterilization, activated neutrophils are highly phosphorylated by the serine residues in p47^phox^, which bind to the p67^phox^ p40^phox^ complex, and then migrate to the plasma membrane to bind to cytochrome b_558_. At this point, an NADPH oxidase is assembled (45).

**Figure 1:**
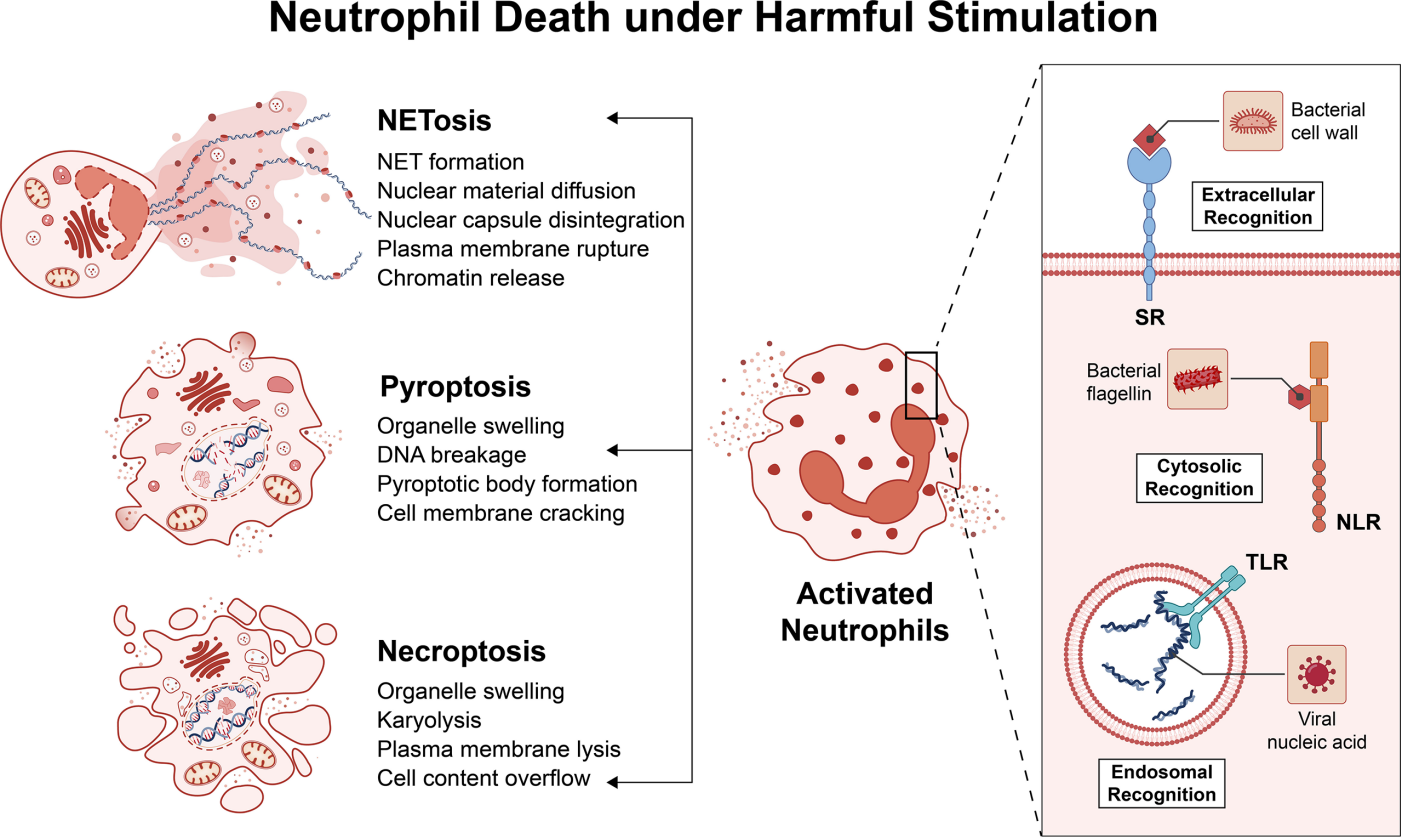
Neutrophil Death under Harmful Stimulation. Neutrophils recognize pathogens through pattern recognition receptors (PRRs). The main PRRs are Toll-like receptors (TLR) or NOD-like receptors (NLR), and there are some other PRRs, such as scavenger receptors (SR). These PRRs can recognize pathogens in the extracellular cytoplasm and endosomes. Neutrophils are activated after recognizing the pathogen and begin to kill the pathogen. The final outcome of an inflammatory environment is often: necrosis, pyroptosis, and neutrophils external traps. These death pathways have different characteristics, but all exacerbate the inflammatory response.

After neutrophils play a bactericidal role at the site of inflammation, they often produce pus. The main forms include necrosis, pyroptosis, and neutrophils external traps (NET) (**Figure 1**) (46). Necrosis is often triggered by intracellular parasites, manifested by the activation of intracellular TLRs, interferon (IFN)-α and granulocyte-macrophage colony-stimulating factor receptors (47). Pyroptosis is activated by the cleavage of Gasdermin D (GSDMD) by the intracellular pathogen inflammasome. Under the action of necrosis and pyroptosis, neutrophils release inflammatory factors, such as interleukin-1β (IL-1β), and the area of tissue damage increases and the degree of damage is more serious (48). The neutrophil extracellular trap is a suicide system that captures and kills microorganisms. The neutrophil extracellular trap is an externalized form of the nucleus and mitochondria, consisting of DNA, histones, and granule proteins. ROS surge activates the proteins arginine deiminase 4 (PAD_4_), neutrophil elastase (NE) and Gasdermin D under the action of the respiratory burst of neutrophils (49). These proteases catalyze the processes of chromatin decondensation, nuclear membrane disassembly, assembly of antimicrobial proteins on chromatin, and cell rupture. Likewise, the cellular contents released by NET will further exacerbate the damage to surrounding tissue. Unlike the first two, NET can also cause autoimmune diseases by exposing cellular endogenous components to immune cells (50).

Conversely, in response to inflammation, neutrophils also die by a non-inflammatory pathway, that is macrophage-mediated efferocytosis. Macrophage uptake of dying neutrophils can rapidly clear cells to prevent secondary necrosis, and can also trigger anti-inflammatory signaling pathways that play an important role in inflammation resolution. If efferent pleocytosis malfunctions, amplifying loops that promote secondary necrosis and exacerbate inflammation (51). It is not difficult to see that macrophages play an important role in the resolution of inflammation. Anti-inflammatory macrophages can secrete various anti-inflammatory mediators (such as IL-10) and express programmed cell death ligand (PD-L), thereby suppressing inflammation (52). However, if pro-inflammatory macrophages cannot complete the transformation to anti-inflammatory macrophages during the initial period of inflammation, a large number of pro-inflammatory macrophages will accumulate, the inflammatory response will continue, and tissue repair will be delayed (53).

## 3 The Connection Between Chronic Inflammation and Chronic Disease

Chronic inflammation is associated with persistent production of pro-inflammatory mediators and persistent activation of pro-inflammatory signaling pathways, and phagocytic-associated inflammasomes, cell differentiation, lipid metabolism, tissue repair, and microbiota contribute to aging-related phenotypes and chronic disease (54).

### 3.1 Inflammation Resolution and Chronic Diseases

Acute inflammation is characterized by a complex but well-coordinated inflammatory response with a resolution period of acute inflammation. Numerous pro-inflammatory cytokines rapidly released by neutrophils characterize the inflammatory environment (TNF and IL-1), and omega-6 pro-inflammatory arachidonic acid (AA)-derived eicosenoic acid (prostaglandins and leukotrienes) (55). These potent pro-inflammatory factors are difficult to identify microbes and their host cells, and thus are prone to irreversible damage to surrounding tissues (4, 56). In an effective acute inflammatory response, damaging factors are rapidly eliminated, while endothelial and immune cells undergo a “lipid mediator switch” that converts pro-inflammatory SPMs into anti-inflammatory prostaglandins, which trigger inflammation subsided, effectively preventing persistent inflammation and tissue necrosis (57). Regression is the process by which inflammation ceases and has historically been seen as a passive process (58). Numerous studies published over the past few years have shown that regression is an active process, manifested by a complex set of mediators that regulate cellular events necessary for inflammatory cell clearance from the site of infection or injury and restoration of tissue function (3, 40). Inflammation can be resolved by removal of inflammatory stimuli, inhibition of proinflammatory signaling, catabolism of proinflammatory mediators, and cellular burial (3). Inflammation may resolve if granulocytes are eliminated during the inflammatory response and the monocyte population (macrophages and lymphocytes) within the tissue returns to pre-inflammatory numbers and phenotypes (59). However, if the inflammatory response persists, a normally healthy immune response can degrade into a dangerous chronic inflammatory disease that can be fatal.

While immune responses are required for successful pathogen clearance, symbiotic contact with commensal microorganisms, wound repair, and overall tissue homeostasis, they can become dysfunctional and initiate persistent responses without resolution phases (4). Persistent stimulation, pro-inflammatory signaling, or damage to pro-catabolic/anti-inflammatory pathways can lead to irreversible inflammatory responses, in which inflammatory cells such as neutrophils and monocytes/macrophages infiltrate the tissue, leaving the tissue chronically inflammatory (31). When these events occur, a normal healthy immune response can deteriorate into a chronic inflammatory state, a hallmark of chronic disease (41). Chronic inflammation has a post-resolution phase involving Ly6Chi inflammatory monocytes (iMOs) and dendritic cells that enhance the adaptive branch of the response, while macrophages in tissues preferentially deplete apoptotic Polymorphonuclear neutrophils (PMNs), thereby connecting innate and adaptive immune system (60–62). The organism tries to establish ‘adaptive homeostasis’ in this new phase, but this may be beneficial in shaping the developmental environment of chronic disease (3). Indeed, adaptive changes are often induced at the expense of many other physiological activities, leading to the formation of non-adaptive traits. In evolution, a balance is established between the beneficial effects of adaptive traits and the undesired effects of non-adaptive traits (4). However, long-term changes in environmental conditions can upset this balance, increase the burden on the body, and lead to the development of chronic diseases. For example, prolonged secondary infection induces autoimmune activation at a lower cost than endogenous antigens, such as those released by apoptotic cells (63). We can conclude that many chronic inflammatory diseases are characterized by persistent acute inflammation combined with failed attempts at adaptive immunity, resulting in immune maladaptation.

### 3.2 Possible Factors Leading to Chronic Diseases

Even though the etiology of some chronic inflammatory diseases is unknown, early detection and treatment are essential to stop their progression. According to Karen T. Feehan et al, the factors that lead to chronic diseases can be roughly divided into (3):

- Aging: Over the years, extensive research has demonstrated an age-related increase in cellular inflammation. Senescent cells secrete large amounts of soluble factors, collectively referred to as the senescence-associated secretory phenotype (SASP), which include large amounts of pro-inflammatory cytokines and chemokines, growth factors, and extracellular matrix (ECM) remodeling enzymes, all of which contribute to A phenomenon known as “aging inflammation” (64). Multiple molecular pathways contribute to the acquisition of cellular senescence-associated secretory phenotypes, including persistent DNA damage response (DDR) (65), unfolded protein response (UPR) (66) and missense nucleic acids (67, 68). By activating NF-κB, these specific intracellular receptors can be detected (69). Sustained activation of NF-κB leads to transcription of numerous genes involved in the control of inflammatory responses, including adhesion molecules such as vascular cell adhesion molecule-1 (VCAM-1) and cytokines such as interleukin (IL-6) and tumor necrosis factor (TNF) (70). Notably, SASP can metastasize senescence by detonating in neighboring cells, a so-called bystander effect, thus creating a pro-inflammatory environment during systemic horizontal spread (71, 72). In addition to the aging-associated secretory phenotype that contributes to senescent inflammation, the thymus produces fewer T cells as we age, reducing our ability to respond to neoantigens and memory for new infections or immunities (73). Increased autoantibodies against self-tissue, memory phenotype T cells release greater amounts of pro-inflammatory cytokines in response to persistent/chronic viral infection (74).
- Self-antigens lead to autoimmune diseases: Chronic inflammation may be induced to a large extent by immune responses to self-tissues. Immune complexes are produced *in situ* or in various organs, including nuclear residues derived from apoptotic cells. The formation of autoantibodies (AAbs) against double-stranded DNA (dsDNA) and other nuclear autoantigens is an important feature of systemic lupus erythematosus, which is easily associated with NETs mentioned above. Strikingly, nearly 100 SLE-associated autoantibodies, including nuclear DNA and nuclear proteins, were detected in NETs (75). Nucleic acid-carrying ICs (immune complexs) may also be phagocytosed by macrophages, releasing pro-inflammatory cytokines. NET components, such as elastase, cathepsin G, and citrullinated histone H3, were also detected in the serum and synovial fluid of RA patients, and these components all have certain damage to the cartilage matrix (76).
- Damage-Associated Molecular Patterns: DAMPs trigger chronic immune responses that alter tissue function, produced and detected by TLRs or NLRs on innate immune cells (77). Obesity is mainly caused by fat cell hypertrophy and excessive calorie intake, and the accumulation of lipids in the bloodstream is the root cause of cardiovascular disease (78). In the pathogenesis of atherosclerosis, early-stage macrophages take up oxidized low-density lipoprotein and other lipids through their TLR ligands, activate NF-κB signaling and trigger the release of inflammatory factors (79). However, the continuous influx of lipoproteins overwhelms the lipid-handling capacity of macrophages and renders macrophage-based lipid clearance systems ineffective (51). Due to the accumulation of lipids in the endoplasmic reticulum membrane, the macrophages are persistently in an inflammatory state, the macrophages are dysfunctional in the efferocytosis of neutrophils, the inflammatory response never enters a regressive state, and chronic inflammation form.
- Microbiota and its secretions: The microbiota in the gut has a significant impact on human health. Chronic inflammation can also be caused by the microbiota due to their ability to affect the gut and surrounding tissues (80). Studies have shown that the diversity of gut microbiota in health and disease and its impact on the environment can vary from protective to pro-inflammatory in animal models of inflammatory bowel disease. Although gut bacteria are known to activate the immune system, persistent inflammation can alter gut microbiota and lead to ecological imbalances (8). During intestinal inflammation, monocyte recruitment was increased, but IL-10 levels were consistently low. In this case, monocytes are insensitive to inflammatory stimuli and acquire the M1 phenotype, secreting a large amount of inflammatory substances such as IL-1β, TNF-α, ROS (59). Furthermore, in IBD, defects in neutrophil migration at sites of inflammation, neutrophil-related oxidative stress, inflammatory factors, disruption of tissue integrity, increased epithelial and vascular permeability, enhanced immune cell recruitment and Inflammation polarizes and hinders wound healing (81). However, due to the complex assemblage of different species, the study of disease phenotypes by specific microbial members is still ongoing.

## 4 miRNAs Regulate Phagocytes in the Pathogenesis of Chronic Diseases

Chronic inflammation is involved in the production of chronic relapsing diseases characterized by excessive activation of the immune response and high levels of autoantibodies, which are autoimmune diseases. Interaction of gene expression and environment plays an important role in the pathogenesis of chronic relapsing disease (82–84). miRNAs are rheostats of gene transcription and are widely involved in innate immune regulation such as phagocytosis, exocytosis, induction of endotoxin tolerance, and cytokine responses (85). Exosomes are extracellular vesicles (MVs) that transport miRNAs between immune cells *via* membrane budding and endocytosis. Notably, some miRNAs are activated during inflammatory responses and can limit excessive immune responses. The imbalance of these miRNAs can lead to uncontrolled production of inflammatory cytokines, which can lead to the occurrence of various diseases. It is not difficult to see that miRNAs are key regulators of innate immune cell development and function and maintenance of immune homeostasis (86). Phagocytes, including neutrophils and macrophages, are the whistleblowers and finishers of the inflammatory response, respectively, and are important in the resolution of inflammation and the development of chronic diseases. In the following sections, we will use these two types of cells as examples to illustrate the regulatory role of miRNAs on them and how they affect chronic diseases.

### 4.1 Neutrophils

Neutrophils are the most abundant innate immune cells in the blood, and during the development of an immune response, neutrophils first reach the site of inflammation/functional damage (87). Myeloblasts in the bone marrow develop into granulosa cells through several morphologically distinct stages, including promyelocytes, myeloid cells, mesenchymal cells, and ribbon cells (88). Granulocyte generation is characterized by differential expression of transcription factors and cyclins, a process controlled by granulocyte-colony stimulating factor (G-CSF) (89). During inflammation, the number of neutrophils at the site of infection damage increases. Normally, neutrophils should be cleared by efferocytosis of macrophages after their mission is complete. This process leads to the down-regulation of the synthesis of inflammatory factors such as IL-23 in cells, thereby reducing the release of G-CSF, and the inflammatory response tends to subside (90, 91).

#### 4.1.1 miRNA and the Function of Neutrophils

Typically, neutrophils are seen as short-lived cells that perform very repetitive roles, such as releasing antimicrobial chemicals, until more specialized cells reach the site of inflammation, enabling a more effective attack. As a result of the activation of multiple cytokines, growth factors, and bacterial products, neutrophils are more complex than initially thought, exhibiting phenotypic and functional diversity and participating in the pathogenesis of health and disease (43, 92). Evidence suggests that neutrophils help activate other immune cells, regulate inflammation and wound healing, which are critical for tissue integrity and the control and resolution of inflammatory processes (92, 93). When exposed to specific stimuli (serum amyloid A17), specific mature neutrophils (which eventually grow into fully formed granules and segmental nuclei) may proliferate outside the bone marrow, prolonging their time in the tissue. Although longer lifespans may allow neutrophils to perform more complex activities in tissues, such as helping resolve inflammation or building adaptive immune responses, their persistence in tissues can damage other cells (94).

Neutrophils are a specialized form of phagocytic cells. When bacteria come into contact with these cells, they eat and destroy the bacteria. Neutrophils engulfing bacteria produce reactive oxygen species (ROS) through an electron transfer system called NADPH, such as O^2-^, HO^-^ and H_2_O_2_ being converted to hypochlorous acid (HOCl) by myeloperoxidase (MPO), which in turn kills bacteria (43, 95). In addition, the bactericidal effect of neutrophils is also reflected in the transport of a variety of different cellular particles with different components and functions (96). Neutrophil granules contain MPO, neutrophil proteases (elastase, cathepsin G, protease 3, azurin) and membrane permeability factors (lysozyme, defensins, bacterial permeability increasing proteins) and are the major germicidal granules (97, 98). This means that neutrophil activation and migration need to be tightly controlled to prevent tissue damage and uncontrolled inflammation.

When discussing inflammatory processes, neutrophils are often viewed as passive components that die and are eliminated over days or weeks, rather than as active participants. They are now known to produce pro-resolution lipid mediators, suggesting that they are actively involved in the resolution-inducing process. GPCRs (G-protein-coupled receptors) and their analogous G-protein-coupled compounds (GPCRs) play important roles in the transport and activation of neutrophils in the *in vivo* environment (99). LXA4 is a lipopolysaccharide (also known as FPR2) that inhibits neutrophil recruitment by binding to its G protein-coupled receptor LXA4R at the end of an acute inflammatory response. By blocking and removing chemokines and cytokines, neutrophils also contribute to the resolution of inflammation (100). Lipolytic mediators such as LXA4, resolvin E1, and protectin D1 promote C-C chemokine receptor type 5 (CCR5) production through apoptotic neutrophils, which then act as functional decoys and scavengers of chemokine (C-C motif) ligand 3 (CCL3) and CCR5 (101).

As a rule, neutrophils are regarded as short-lived cells that perform a very recurring role, such as releasing antibacterial chemicals, until more specialized cells arrive at the inflammatory site, allowing for more effective attacks. Half-lives in mice and humans are 1.5 and 8 hours, respectively (102, 103).To ensure that neutrophils are present at the site of inflammation, they are activated, and their life span is enhanced severalfold during the inflammatory process (104). Neutrophils are more complex than first thought. They exhibit phenotypic and functional diversity and are involved in the pathogenesis of both health and disease (43, 92), as a result of activation by a variety of cytokines, growth factors, and bacterial products (105). There is evidence that neutrophils contribute to the activation of other immune cells, regulation of inflammation, and wound healing, which is crucial for tissue integrity and ordinance and resolution of the inflammation process (92, 93). Upon exposure to specific stimuli, such as serum amyloid A17, particular mature neutrophils (which eventually grow into fully-formed granules and segmented nuclei) may multiply outside the bone marrow, lengthening their time in the tissue. Even though a more extended lifespan may allow neutrophils to perform more complex activities in the tissue, such as helping to resolve inflammation or establishing an adaptive immune response, their continuing presence in the tissue may harm other cells (94).

Finally, the treatment of apoptotic neutrophils is a critical step in addressing inflammation, which is carefully regulated by the expression of an “eat me” signal that initiates an anti-inflammatory program in phagocytes (106, 107). Indeed, the recognition and uptake of apoptotic neutrophils can influence the phenotype of macrophages (106), and macrophages themselves also polarize towards an anti-inflammatory type when they perform efferocytosis on apoptotic neutrophils, release anti-inflammatory factors and promote tissue repair (108, 109). Thus, neutrophils are part of the cellular cascade that coordinates the resolution of inflammation. They are important for shutting down the inflammatory response early and preventing the development of chronic inflammation.

One of the most effective ways neutrophils fight infection and tissue damage is through their immune system. Neutrophils are a specialist form of phagocyte. These cells can eat and destroy bacteria when they come into touch with them. An electron transfer system known as NADPH is a multi-protein electron transfer system that can be assembled and activated to generate reactive oxygen species (ROS) such as O^2-^, HO^-^, and H_2_O_2_ (43, 95). Myeloperoxidase(MPO) converts H_2_O_2_ to hypochlorous acid (HOCl). Neutrophils transport a variety of different cell particles with distinct components and functions (96). The neutrophil granules contain MPO, neutral protease (elastase, cathepsin G, protease 3, and azurin), and membrane permeability factors (lysozyme, defensin, and bacterial permeability-increasing protein), which are the main bactericidal granules (97, 98). This means that neutrophil activation and migration need to be strictly controlled in order to prevent tissue damage and inflammation from becoming out of control. GPCRs (G protein-coupled receptors) and their analogous G protein-coupled compounds (GPCCs) play an essential role in the trafficking and activation of neutrophils *in vivo* environments (99).

miRNAs play key roles in cellular processes such as granulocyte proliferation, activation, and apoptosis. Mef2c is an important regulator of granulocyte development. Johnnidis et al. found that miR-223-deficient mice had neutrophil hyperactivity and hyperinflammatory function caused by direct targeting of transcription factor MEF2C, suggesting that miR-223 is a negative regulator of granulocyte production and inflammatory response (110). These findings suggest that miR-223 acts as a regulator of granulocyte activation, effectively suppressing pathogenic immune responses (111). The overexpression of miR-21 is closely related to the activation of granulocytes (112). Elevation of miR-199 reduces neutrophil chemotaxis and migration by inhibiting the cyclin-dependent kinase 2 (Cdk2) pathway, ultimately reducing the inflammatory response (113). MiR-9 is a component of the feedback loop of granulocyte-induced inflammation, and miR-9 inhibits the synthesis of NF-κB by regulating the TLR4 pathway, thereby activating neutrophils (114). The role of miR-155 in granulocytes has been revealed *in vitro* and *in vivo*. MiR-155 is essential for granulocyte proliferation by regulating SH2-containing inositol 5’-phosphatase 1 (SHP1). An animal model study showed that elevated miR-155 produces myeloproliferative disorders, suggesting that miR-155 is critical for maintaining the balance of innate immune cells (96, 115).

#### 4.1.2 miRNA and NETs

Under inflammatory conditions, neutrophils release a network of complexes consisting of chromatin DNA, histones, and granule proteins into the extracellular environment, leading to extracellular death, a structure known as a neutrophil extracellular trap (116). Granular proteins that have been identified in NET include antimicrobial proteins (such as lactoferrin, cathepsin G, defensins, LL37, and bacterial permeability-increasing proteins), proteases (such as neutrophil elastase, protease 3 (PR3), and gelatin enzymes) or enzymes responsible for the production of reactive oxygen species such as myeloperoxidase (MPO) (116). Recent evidence suggests that NETs and their components may be detrimental to host tissues and have contributed to the development of many non-infectious diseases (117), such as atherosclerosis (118), systemic lupus erythematosus (119), vasculitis (120), and thrombosis (121, 122). Antineutrophil cytoplasmic antibody (ANCA)-associated vasculitis (AAV) is a systemic necrotizing vasculitis of small vessels characterized by the production of antineutrophil cytoplasmic antibodies against neutrophil cytoplasmic proteins (ANCA) (123, 124), targeting some NET components such as MPO, PR3 and neutrophil elastase (125). Methods such as DNase and NET-DNA targeting using NET-associated proteins have demonstrated that inhibiting the production of NETs can prevent tissue damage (126).

**Figure 2:**
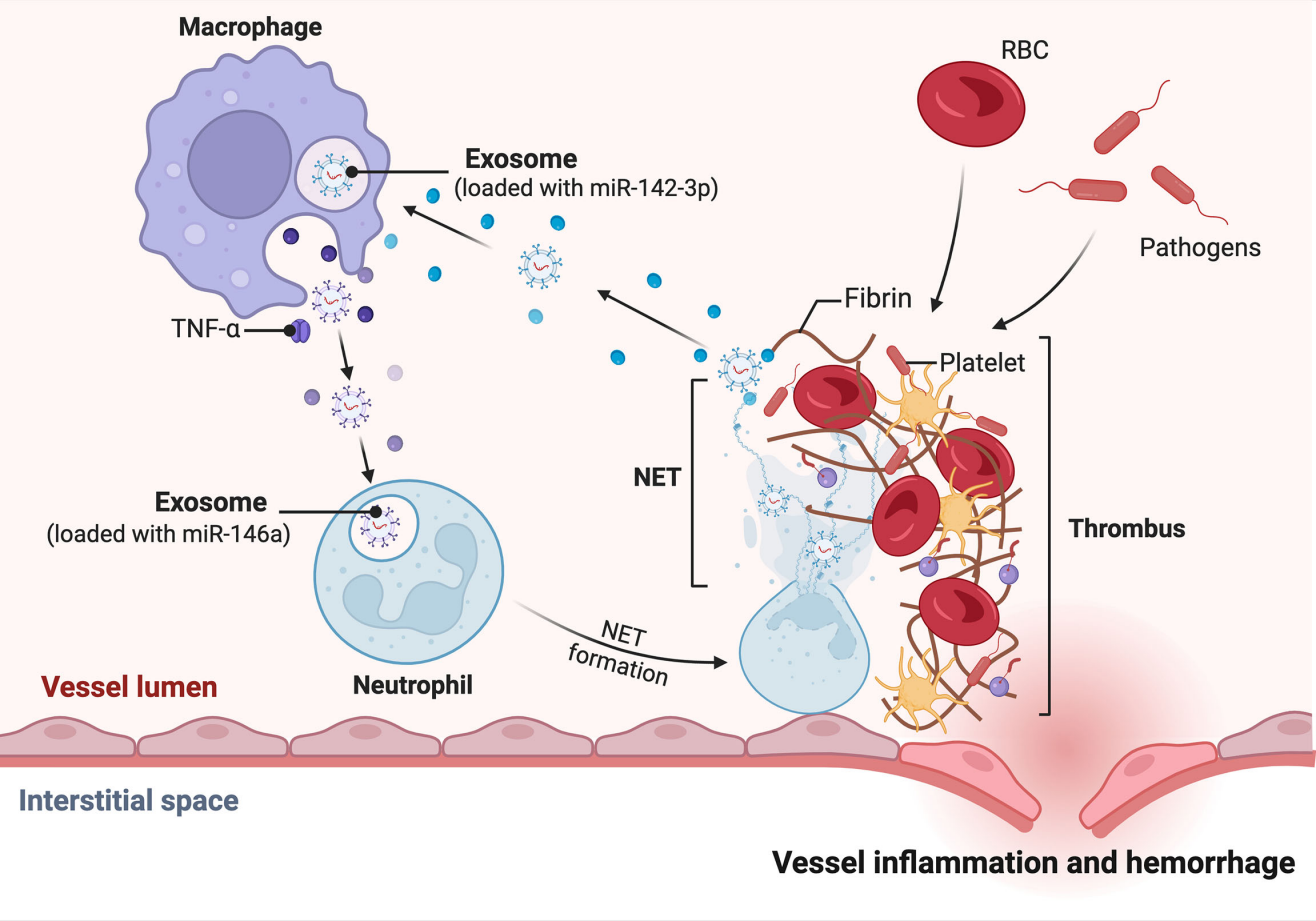
miRNA-mediated regulation of inflammatory factors by NET released in neutrophils. Under the influence of the vascular inflammatory environment, neutrophils, for example, mature macrophages release exosome-loaded miR-146, which stimulates neutrophil development and maturation to form NETs. The NETs supernatant contains miRNA, such as miR-142-3p. miR-142-3p targets macrophages to release more pro-inflammatory factors, which in turn accelerate the formation of a pro-inflammatory environment.

Recent studies have shown that NET and miRNA are very closely related. Linhares-Laseda et al. are the first to demonstrate the presence of NET-associated miRNA vectors and miRNAs in NET-enriched supernatants (NET-miRs). This provides a new class of molecules and a new protein platform that can be created and delivered in NETs. Their research revealed a novel role for NET in cellular communication, facilitating the transport of miRNAs from neutrophils to neighboring cells. NET, as a negative feedback loop, reduces hyperreactivity and maintains normal regulation of inflammatory responses (**Figure 2**). In monocytes/macrophages, the protein kinase C (PKC) pathway is involved in adhesion/migration, M1/M2 polarization, TLR activation and inflammatory cytokine production (127). It has been shown that the role of miRNA-142-3p may also affect these activities, suggesting that NET may allow extensive control of surrounding cellular functions through the release of miRNAs. Specifically, miRNA-142-3p carried by NET downregulates protein kinase Cα (PKCα) and regulates TNF-α production in macrophages when NET interacts with macrophages (128–130). Not only that, miRNAs released by surrounding cells *via* exosomes can also affect neutrophil NETosis formation. In Yong-Zhanggan et al.’ s study, exosomal miR-146a produced by oxLDL-treated macrophages stimulated ROS and NET production and worsened atherosclerosis by targeting SOD2 (131). The study by Reyes-García AML et al. showed that NET represents an important relationship between inflammation and thrombosis, and that both NET components DNA and H4 lead to increased HNF4A mRNA expression, which may suggest that it is partly involved in the Coagulation factor regulation. They determined that H4 induced decreased expression of specific miRNAs in the miR-17/92 cluster, which partially explains why H4 induced increased TF expression (132).

#### 4.1.3 miRNAs Regulate Neutrophil-Involved Chronic Diseases

Although it has been traditionally believed that the primary role of neutrophils is to effectively eliminate extracellular pathogenic factors, it is not surprising that neutrophils play an important role in the pathogenesis of many diseases based on the recent discovery of a wide range of neutrophil functions. Some results indicate that neutrophils have become an important determinant of chronic inflammation (133). In the following section we would discuss about the role of miRNAs in regulating neutrophils in chronic diseases such as sepsis, asthma, systemic lupus erythematosus and so on (**Table 1**).

**Table 1:** miRNAs regulate neutrophil-involved chronic diseases.

miRNA	Expression level	Target	Function	Chronic diseases	Ref.
miR-21	↑	PGE 2/IL-10	pro-inflammation	sepsis	(134)
miR-223-3p	↓	MKNK1	pro-inflammation	sepsis	(135)
miR-146a	↑	NF-κB	pro-inflammation	sepsis	(136, 137)
		SOD2	promote NETosis		
miR-887-3p	↑	IL-1β	pro-inflammation	sepsis	(138)
		VCAM-1			
miR-155	↑	NF-κB	pro-inflammation	sepsis	(139)
miR-let-7b	↑	TLR4	anti-inflammation	sepsis	(140)
		NF-κB			
miR-223	↑	NLRP3/IL-1β	anti-inflammation	asthma	(141)
hsa-miR-223-3p	↑	TLR/Th17	endoplasmic reticulum stress	asthma	(142)
miR-199a-5p	↑	WNT2	inhibit lung regeneration	asthma	(143)
		WNT4			
miR-629-3p	↑	IL-8	pro-inflammation	asthma	(144)
miR-4512	↓	TLR4	promote NETosis	SLE	(23)
		CXCL2			
miR-let-7b	↑	TLR-7	pro-inflammation	SLE	(145)
miR-125a	↓	IL-16	pro-inflammation	SLE	(146)
miR-223-3p	↑	GM-CSF	anti-inflammation	COPD	(147)
		TRAF4			
miR-1285	↑	SP11	pro-inflammation	IBD	(144)
			inhibit tissue repair		
miR-23a	↑	Lamin B1	inhibit tissue repair	IBD	(148)
miR-155		RAD51	pro-inflammation		
miR-223	↑	IL-18	anti-inflammation	AOSD	(149)

PGE 2, prostaglandin E2; IL-10, interleukin-10; MKNK1, mitogen-activated protein kinase interacting serine/threonine kinase 1; NF-κB, nuclear factor kappa light-chain enhancer of activated B cells; SOD2, manganese superoxide dismutase, superoxide dismutase 2; VCAM-1, vascular cell adhesion molecule-1; TLR4, toll-like receptor 4; NLRP3, Nucleotide-binding oligomerization domain, leucine-rich repeat and pyrin domain-containing 3; Th17, helper T cell 17; WNT, Wingless-Type MMTV Integration Site Family; CXCL2, Chemokine(C-X-Cmotif) ligand10; GM-CSF, granulocyte monocyte-colony stimulation factor; TRAF4, (TNF) tumor necrosis factor-receptor-associated factor 4; SP11, S-locusprotein 11; RAD51, a homologous recombination regulator homologous recombination; SLE, Systemic lupus erythematosus; COPD, chronic obstructive pulmonary disease; IBD, inflammatory bowel disease; AOSD, Adult-onset Still’s disease. ↑ Represents expression level rises. ↓ Represents expression level decreases.

##### 4.1.3.1 Sepsis

When ill individuals encounter an inappropriate immune response, it can lead to exacerbations of sepsis, and the cardiovascular system is a susceptible system to sepsis (150). On the anti-inflammatory side, miR-21 drives an overwhelming inflammatory response by indirectly inhibiting the expression of the anti-infective mediator prostaglandin E2(PGE 2)/IL-10 (134). MiR-let-7b directly targets toll-like receptor 4 (TLR4) and nuclear factor κB (NF-κB), reducing interleukin-6 (IL-6), IL-8, tumor necrosis factor alpha (TNF-α) and other pro-inflammatory factors, up-regulate the anti-inflammatory factor IL-11 (140). In terms of pro-inflammatory, up-regulation of miR-146a, miR-887-3p, miR-155, and down-regulation of miR-223-3p play important roles. miR-146a modulates inflammatory responses by inhibiting the Toll-like receptor/NF-κB axis and sod2 and modulates NET formation by altering its senescence phenotype (136, 137). MiR-887-3p released by neutrophils increases endothelial release of chemokines and promotes transendothelial leukocyte migration (138). Furthermore, neutrophils promote vascular inflammation and atherosclerosis by delivering miR-155-carrying microvesicles to disease-prone areas (139). Downregulation of miR-223-3p promotes the expression of mitogen-activated protein kinase (MAPK)-interacting serum/threonine kinase 1 (MKNK 1), which regulates the abundance of neutrophil-expressed inflammatory factors involved in sepsis (135).

##### 4.1.3.2 Asthma

Asthma is a chronic respiratory disease. Airway obstruction in asthma includes bronchial smooth muscle spasms and different degrees of airway inflammation, which are characterized by edema, mucus secretion, and inflow of various inflammatory cells (151). Expression of miR-223 in neutrophils inhibits the NLRP3/IL-1β axis, reduces airway inflammation, and reduces NLRP3 (Nucleotide-binding oligomerization domain, leucine-rich repeat and pyrin domain-containing 3) levels and IL-1β release (141). Has-miR-223-3P, a neurotropic miRNA, regulates TLR/Th17 signaling and endoplasmic reticulum stress by inhibiting the TLR/Th17 pathway (142). The up-regulation of miR-199a-5p in neutrophils is negatively correlated with lung function (143). MiR-629-3p damages the bronchial epithelium by inducing IL-8 mRNA expression and promoting inflammatory response (144). In addition, miR-26a, miR-146a, and miR-31 are also related to the levels of interleukin-5 (IL-5), IL-8, IL-12, and tumor necrosis factor-α (TNF-α) (152).

##### 4.1.3.3 Systemic Lupus Erythematosus

Systemic lupus erythematosus is a chronic autoimmune disease characterized by the loss of self-tolerance and the formation of nuclear autoantigens and immune complexes. The disease has a wide range of manifestations, which can involve multiple organ system inflammations. The course of the disease is chronic or relapsed and alleviated, leading to significant morbidity and even mortality (153). In SLE, the down-regulation of miR-4512 in neutrophils promotes the expression of TLR4 and CXCL2, which has the function of promoting the formation of NETs (23). As a TLR-7 agonist, miR-let-7b appears in pro-inflammatory neutrophils (low-density granulocytes (LDGs)) NET of SLE and plays a role in inducing vascular cell pro-inflammatory response (145). In addition, the down-regulation of miR-125a weakened the original inhibitory effect on IL-16 gene, and the up-regulation of IL-16 expression directly acts on lung epithelial cells, thereby significantly enhancing the expression of neutrophil chemokines, leading to lung injury (146).

##### 4.1.3.4 Other Chronic Diseases

In inflammatory bowel disease (IBD), miR-23a and miR-155 enhance the deleterious effects of neutrophils by targeting lamin B1 and RAD51 (a homologous recombination regulator), inhibiting tissue healing responses (148). Neutrophil-derived traces (NDTR) are membrane-derived vesicles produced by neutrophil migration toward inflammatory foci and contain pro-inflammatory miRNAs, such as miR-1285. MiR-1285 promotes intestinal inflammation and inhibits tissue repair by targeting the S-locusprotein 11 (SP11) gene. Other miRNAs in NDTR, including miR-1260, miR-4454, and miR-7975, have similar utility (144). In atherosclerosis, miR-146a (a brake of inflammatory response) is downregulated, thereby increasing NETosis and increasing thrombotic risk (137). In rheumatoid arthritis, citrullinated protein antigen and TNF-α decreased the expression of many miRNAs and their biogenesis-related genes, such as miRNA-223, miRNA-126 and miRNA-148a, thereby increasing their potential mRNA target. These miRNAs are mainly associated with migration and inflammation in synovial fluid neutrophils (154).In chronic pulmonary disease (COPD), miR-223-3P suppressed Granulocyte monocyte-colony stimulation factor (GM-CSF) secret and gene expression of the pro-Inflammatory transcription factor traf4, which is related to neutrophilic inflammation (147). In Adult-onset Still’s disease (AOSD), the expression of miR-223 in neutrophils is suppressed (149). In IBD, miR-23a and miR-155 can enhance the harmful effects of polymorphonuclears (PMNs) and inhibit the tissue healing response (148).

### 4.2 Macrophages

Macrophages are ubiquitous in our body, and most tissue-resident macrophages are seeded in the yolk sac in embryonic form for long-term self-renewal (155). Tissue macrophages are specialized according to the microenvironment of the tissue in which they live and have specific functions. Therefore, they are not only immune cells, but also participate in the formation of living tissues through specialized auxiliary functions, such as osteoclasts in bone (156), macrophages in intestinal muscularis (157), and small keratinocytes in brain tissue (158). The remaining macrophages were derived from monocyte-macrophages. Monocytes are present in the blood circulation and have a high degree of functional plasticity, providing the necessary support for their involvement in the initiation and subsequent resolution of inflammatory responses (4, 159). In an inflammatory response, damaged infected areas can chemotactically recruit mature monocytes, expose them to several cytokines and bacterial products, and differentiate into macrophages. They participate in inflammatory processes together with tissue-resident macrophages to maintain the macrophage pool in the tissue (160, 161).

Dynamic regulation of complex gene networks and signaling cascades that control macrophage polarization, priming, and plasticity through multiple layers of regulation of gene expression (162). Transcription and translation are complex processes that are tightly regulated and strongly influence cellular function. Specific miRNA subsets induced by different microenvironmental signals have been shown to modulate transcriptional output to obtain distinct macrophage activation patterns and polarization states, ranging from M1 phenotype to M2 phenotype (163), affecting multiple macrophages biology, such as monocyte differentiation and development, macrophage polarization, infection, inflammatory activation, cholesterol homeostasis, cell survival, and phagocytosis (164).

#### 4.2.1 miRNA and the Plasticity of Macrophages

Macrophages are an important plastic cell. The local microenvironment can make macrophages directional polarization, from one phenotype to another phenotype (165). Macrophages are heterogeneous, and their phenotype and function are regulated by the surrounding microenvironment. Macrophages are generally divided into two distinct subpopulations: a) M1 macrophages (typically activated macrophages), induced to differentiate by lipopolysaccharide (LPS) alone or in combination with Th1 cytokines such as IFN-γ and TNF-α, secrete high levels of pro-inflammatory cells Factors, such as interleukin-1β (IL-1β), IL-6, IL-12, and cyclo-oxygen-ase-2 (COX-2), have pro-inflammatory effects. M1 macrophages have potent antibacterial and antitumor activities and are able to mediate ROS-induced tissue damage while impairing tissue regeneration and wound healing (166–169). b) M2 macrophages (alternately activated macrophages), which have anti-inflammatory and immunomodulatory effects, are polarized by Th2 cytokines (such as IL-4, IL-13) and produce anti-inflammatory cytokines (such as IL- 10, TGF-β). Exposure of M2 macrophages to the M1 signaling environment results in “repolarization” or “reprogramming” of differentiated M2 macrophages and vice versa (165, 168, 170, 171). M1 macrophages and M2 macrophages have distinct functional and transcriptional profiles, and the balance of polarization between them determines the fate of inflamed or injured organs. When in an infection or inflammatory response, macrophages first exhibit the M1 phenotype to resist the stimulation to release anti-inflammatory factors such as TNF-α and IL-1β. At this time, M2 macrophages secrete a large amount of IL-10 and TGF-β to inhibit inflammation, promote tissue repair, angiogenesis, and maintain environmental stability (51).

MiRNA-125, miR-127, miRNA-146, miRNA-155 and miRNA-let-7a/f were involved in the polarization of M1 macrophages, while miRNA-9, miRNA-21, miRNA-146, miRNA-147 and miR-223 regulates the polarization of M2 macrophages. Compared with miR-155 and miR-142-3p, miRNA-let-7a reduced macrophage proliferation. Macrophage apoptosis is negatively regulated by miR-21 and miRNA-let-7e (172–174). GATA binding protein 3(GATA3) is targeted by miR-720, whereas BCL6 is targeted by miR-127 and miR-155, all of which induce M1 polarization. The polarization of M2 macrophages is dependent on these two miRNAs (175, 176). On the other hand, overexpression of miR-720 reduces M2 polarization (175). There is evidence that miR-127 and miR-155 increase pro-inflammatory cytokines, and that M2 macrophages can be transformed into M1 macrophages by overexpression of miR-155 (22, 176–178). MiR-146a increases the expression of M2 marker genes (such as CD206) in peritoneal macrophages and decreases the expression of M1 phenotypic markers, resulting in M2 polarization of macrophages (such as IL-12) (179).

**Figure 3:**
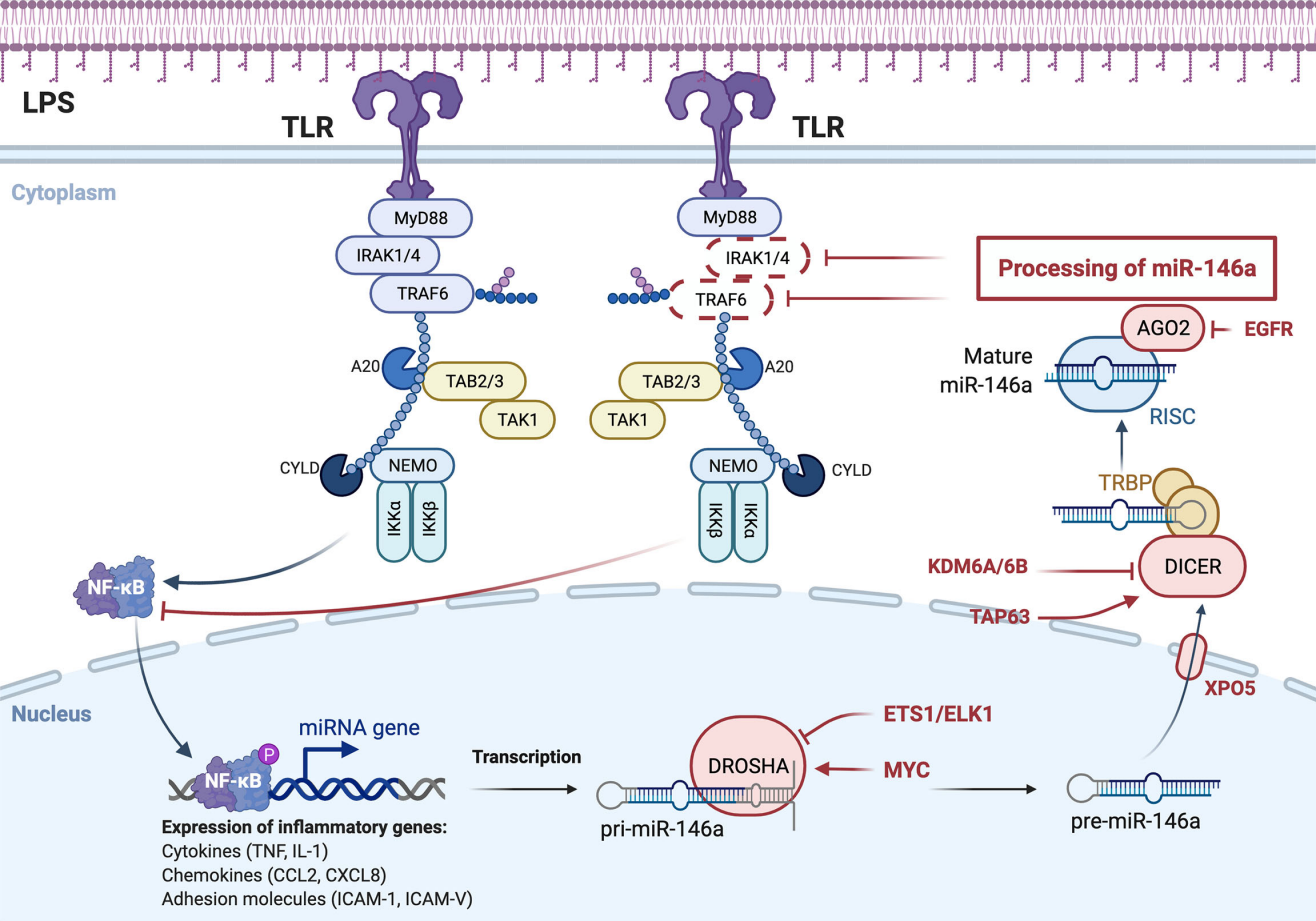
miRNA-mediated negative feedback loop of macrophage polarization to M1 type. TLR on the macrophage cell membrane, stimulated by bacterial LPS, activates NF-κB pathway, which in turn promotes the formation of miR-146a, and the formation of miR-146a inhibits NF-κB pathway by inhibiting IRAK1 and TRAF6. Expression and function are two distinct concepts for the same miRNA family members.

The role of miRNAs in regulating macrophage polarization allows it to influence the duration and intensity of the innate immune response, which helps prevent excessive macrophage inflammation. miRNAs may transform macrophages from pro-inflammatory to anti-inflammatory by affecting the expression of immune proteins (180, 181). MiR-146a and miR-155 are the earliest expressed miRNAs in LPS-induced macrophages and are controlled by NF-κB (182, 183). Numerous studies have confirmed that there is a negative feedback loop in the production of miRNAs in the NF-κB pathway (**Figure 3**). The NF-κB pathway is inhibited by miR-146a, which increases transcription of two distinct miR-146a targets: the adaptor proteins TNF receptor associated factor 6 (TRAF6) and interleukin-1 receptor associated kinase 1 (IRAK1) (182). MiR-155, a pro-inflammatory miRNA, is also involved in this negative feedback regulation, rapidly increasing NF-κB expression in macrophages using TLR ligands and type 1 interferons (184). Notably, miR-155 is a key component of various feed forward networks that regulate the length and intensity of inflammatory responses (185, 186).

Members of the same miRNA family may have completely opposite regulatory effects. LPS-activated macrophages express distinct miR-125a and miR-125b, which play an antagonistic role in cellular inflammatory responses. In contrast to miR-125a, the level of miR-125b was decreased at an early stage in LPS-induced macrophages. Enhanced expression of miR-125b induces stronger IFN-γ responses and maintains activation of pro-inflammatory cells by targeting innovation and research focus (IRF4), thereby promoting M2 macrophage polarization (187, 188). Similarly, miR-146a and miR-146b may act as a relay system to buffer TLR4 trigger-induced expression of proinflammatory genes (189).

#### 4.2.2 miRNAs Regulate Macrophage-Involved Chronic Diseases

Macrophages play a central role in the innate immune response and are the link between the innate and adaptive immune responses. Macrophages directly neutralize pathogens by phagocytosis and secrete chemokines and cytokines to coordinate the response of other immune cells (such as neutrophils and lymphocytes) and the stroma (159). Macrophages have a variety of functions, including: a) phagocytosis and killing, b) antigen presentation, c) mediating inflammatory responses, which include interleukin-1 (IL-1), IL-6, and TNF-α with different types of cytokines to achieve (190, 191), d) Tissue repair, regeneration and fibrosis (53), e) lipid metabolism (192).

As stated in “The Doctor’s Dilemma” (Act 1): “There is really only one truly scientific cure for all diseases, and that is to stimulate phagocytes.” Macrophages are key in chronic inflammation and related pathological processes cell (193). While macrophages are critical for effective control and clearance of infections, clearance of pathogens and dead cells, and promotion of tissue repair and wound healing, they may also cause tissue damage and pathological changes during infections and inflammatory diseases (194). What is more needed now is to calm them down so that the inflammatory response can be addressed. M2 macrophages play a key role in the resolution of inflammatory responses. Phagocytic debris, damaged or dead cells, and apoptotic neutrophils are essential functions of M2 macrophages in this process. During tissue breakdown, macrophages are the main source of lipid mediators and produce anti-inflammatory cytokines, and IL-10 and TGF-β are involved in tissue breakdown (195, 196). After being stimulated by the extracellular environment, macrophages can adjust their own miRNA secretion levels to adapt to the environment. In addition, miRNAs secreted by macrophages can also be transported to other cells through extracellular vesicles, thereby regulating the functions of these cells. The following summarizes the role of miRNAs in regulating macrophages in some chronic diseases (**Table 2**).

**Table 2:** miRNAs regulate macrophage-involved chronic diseases.

miRNA	Expression level	Target	Function	Chronic diseases	Ref.
miR-342-5p	↑	Akt1	pro-inflammation	in early AS	(197)
miR-155	↑	CSF-1	inhibit macrophage lesion	in early AS	(178)
miR-92a	↑	KLF2	pro-inflammation	in early AS	(198)
miR-383	↓	Parg	promote macrophage survival	in early AS	(199)
miR-10a	↑	LCoR	promote lipid metabolism	in early AS	(200)
miR-10b	↑	ABCA1	reduclate plaque	in advanced AS	(201)
miR-155	↑	Bcl6	pro-inflammation weaken efferocytosis	in advanced AS	(22)
miR- 302a	↑	ABCA1	promote lipid metabolism	in advanced AS	(202)
miR-155	↑	CEH	inhibit foam cell	AS	(203)
miR-17-5p	↓	NF-κB	promote lipid metabolism	AS	(25)
miR-34a	↑	ABCG1 liver X receptor α	pro-inflammation	AS	(204)
miR-146a	↑	SOD2	pro-inflammation	AS	(131)
miR-140a	↑	IL-10	pro-inflammation	AS	(205)
miR-21-3p	↑	PTEN	promote tissue repair	AS	(206)
miR-34a	↑	KLF4	pro-inflammation promote insulin resistance	Obesity	(207)
				T2DM	
miR-210	↑	NDUFA4	promote insulin resistance	Obesity	(208)
				T2DM	
miR-690	↑	Nadk	insulin sensitizer	Obesity	(209)
				T2DM	
miR-467a-5p	↑	THBS1	prevent insulin resistance	Obesity	(210)
				T2DM	
miR-505-3p	↓	RUNX1	pro-inflammation	Obesity	(211)
				T2DM	
miR-29	↑	TRAF3	pro-inflammation	Obesity	(212)
				T2DM	
miR-712	↓	LRRK2	anti-inflammation	Obesity	(213)
				T2DM	
miR-128-2	↑	ABCA1	promote lipid metabolism	Obesity	(214)
		ABCG1		T2DM	
		RXRa			
miR-33a	↑	ABCA1	inhibit lipid metabolism	Obesity	(24)
		ABCG1		T2DM	
miR-221-3p	↓	JAK3	pro-inflammation	RA	(215)
miR-29b	↑	HBP1	pro-inflammation	RA	(216)
miR-132	↑	COX2	promote osteoclastogenesis	RA	(217)
miR-574-5p	↑	TLR 7/8	promote osteoclastogenesis	RA	(218)
miR-20a	↑	RANKL	inhibit osteoclastogenesis	RA	(219)
miR-6089	↑	TLR4	inhibit osteoclastogenesis	RA	(220)
miR-148a	↓	GP130	pro-inflammation	IBD	(221)
		IKKα			
		IKKβ			
		IL1R1 TNFR2			
miR-590-3p	↑	LATS1	anti-inflammation promote tissue repair	IBD	(222)
miR-378a-5p	↑	NLRP3	anti-inflammation promote tissue repair	IBD	(223)
miR-142-5p	↑	SOCS1	promote fibrosis	liver cirrhosis	(26)
miR-130a-3p	↓	PPARγ	promote fibrosis	liver cirrhosis	(26)
miR-4512	↓	TLR4 CXCL2	pro-inflammation	SLE	(23)
miR-20a	↑	IL-18	anti-inflammation	AOSD	(149)
miR-181b	↑	PKCδ	regulate macrophage polarization	Myocardial infarction	(224)

Akt1, serine/threonine protein kinase 1; CSF-1,colony-stimulating factor-1; KLF2,Krüppel-like factor 2; Parg, poly(ADP-ribose)-glycohydrolase; LCoR, ligand-dependent nuclear receptor corepressor; Bcl6, B-cell lymphoma 6 protein; RXRα, Retinoid X receptors α; ABCA1, ATP-binding cassette transporter A1; ABCG1, ATP-binding cassette subfamily G member 1; JAK3, Janus kinase 3 tyrosine-protein kinase; COX, Cyclooxygenase; IKK, inhibitor of nuclear factor kappa-B kinase; PTEN, phosphatase and tension homologue deleted from chromosome 10; KLF4, Krüppel-like factor 4; NDUFA4, NADH dehydrogenas, ubiquinone 1 alpha subcomplex 4; THBS1, thrombospondin 1; IL1R1, interleukin 1 receptor type 1; TNFR2, TNF receptor superfamily member 1b; TRAF3,TNF-receptor-associated factor 3; NF-κB, nuclear factor kappa light-chain enhancer of activated B cells; HBP1, the high-mobility group box-containing protein 1; TLR,Toll-like receptor; RANKL, receptor activation of nuclear factor-κB ligand; NLRP3, NOD-like receptor family, pyrin domain-containing 3; SOCS1,suppressor of cytokine signaling 1; PPARγ, peroxisome proliferator-activated receptor γ; PKCδ,protein kinase C δ; AS, atherosclerosis; T2DM, diabetes mellitus type 2; AR, rheumatoid arthritis; IBD, inflammatory bowel disease; SLE, Systemic lupus erythematosus; AOSD, Adult-onset Still’s disease.

##### 4.2.2.1 Atherosclerosis

Vascular wounds heal slowly when stimulated by hyperglycemia, hypertension or nicotine. In the early stage of atherosclerosis, the vascular injury area becomes an inflammatory microenvironment, and inflammatory cells (such as neutrophils and macrophages) gather, making the vascular endothelium vulnerable to injury. In the mid-stage, lipids rich in blood impair the regenerative capacity of endothelial cells and cause the accumulation of moxLDL in macrophages, transforming into foam cells. In the advanced stage, it is difficult for the vascular wound to heal. The constant influx of lipoproteins causes the lipid clearance system of macrophages to fail. Cholesterol accumulation in the endoplasmic reticulum of macrophages leads to the same effects as activation of toll-like receptors 2 (TLR2) and 4 (TLR4) and inflammatory activation of macrophages (12). It can be seen that in the process of AS, macrophages are gradually damaged, resulting in secondary necrosis of apoptotic cells and aggravation of inflammation.

The regulation of macrophages by miRNAs exists in various stages of atherosclerosis. In the early stage of trauma, monocytes-macrophages are rapidly recruited by inflammatory factors and differentiate into large numbers of macrophages. Under the influence of a diminished generation of nitrotyrosine, miR-342–5p is up-regulated in macrophages and induces macrophages to produce pro-inflammatory factors (such as Nos2, IL-1β, and IL-6) by an Akt (protein kinase B) 1- and miRNA-155-dependent pathway. Up-regulation of miR-342-5p also results in decreased expression of Bmpr2 (bone morphogenetic protein receptor, type II). Bmpr2 mRNA may regulate the synthesis of inflammatory mediators in macrophages by binding to miR-342-5p, which competes with Akt1 (197). MiR-92a directly targets Krüppel-like factor 2 (KLF2) to increase the expression of KLF

2, endothelial nitric oxide synthase, and thrombomodulin (198). The expression of miR-155 in macrophages is increased. miR-155 can inhibit the proliferation of macrophages and reduce the content of diseased macrophages by targeting colony-stimulating factor-1 (178). The down-regulation of miR-383 in macrophages also has a similar effect. The down-regulation of miR-383 reduces energy consumption and increases the cell survival rate of bone marrow-derived macrophages by reducing the inhibition of the poly (ADP-ribose)-glycopyrrolate gene (PARG) (199). In addition, miR-10a is upregulated in macrophages and mediates Dicer lipolytic and anti-inflammatory effects by inhibiting ligand-dependent nuclear receptors and promoting fatty acid oxidation (200). In the late stage, under the induction of Free Cholesterol-Induced Macrophase Apoptotic Cells (FC-AM), miR-10b in resident peritoneal macrophages (RPM) was up-regulated to reduce the expression of ABCA1 in RPM, thereby reducing the size of late plaques and enhancing the stability of plaques (201). Stimulated by a variety of inflammatory mediators, including mildly oxidized low-density lipoprotein (moxLDL), miR-155 reduces the anti-infectious signaling proteins (Bcl-6) and phosphorylated-stat-3, thereby enhancing the expression of inflammatory mediators in macrophages (such as CCL2) and impairing efferocytosis. In the hypercholesterolemia environment, miR-302a is up-regulated to inhibit the expression of ATP-binding cassette transporter A1 (ABCA1), to stimulate the lipid-cleaning function of macrophages with cholesterol accumulation in plaques (202).

In addition, miR-17-5p, miR-140a, and miR-146a played a pro-inflammatory role in AS. SNHG16 is up-regulated and inhibits the expression of miR-17-5p, the proliferation, infectious factors, and NF-κB signaling factors are increased in macrophages, thus promoting the inflammatory response in AS patients and the proliferation of THP-1 macrophages (25). Monocyte-derived miR-140a inhibits IL-10 expression, enhances the pro-inflammatory capacity of ox-LDL-stimulated differentiated macrophages, and reduces IL-10-mediated anti-inflammatory response (205). MiR-146a, on the other hand, promoted the release of ROS and NETs by inhibiting SOD2 (131). In terms of anti-inflammatory, miR-155 from THP-1 macrophages inhibits foam cell formation and enhances cholesterol efflux (203). miR-34a regulates macrophage cholesterol efflux and reverses cholesterol transport by inhibiting ATP-binding cassette subfamily G member 1 (ABCG1) and liver x receptor α (204). In the aspect of tissue repair, exosomes derived from nicotine-treated macrophages inhibit phosphatase and tension homologue deleted from chromosome 10 (PTEN) by releasing miR-21-3p to promote the migration and proliferation of vascular smooth muscle cells (VSMCs) (206).

##### 4.2.2.2 Obesity and Type 2 Diabetes Mellitus

Type 2 diabetes mellitus (T2DM) is a chronic low-grade inflammatory disease characterized by insulin resistance (IR) and pancreatic β -cell dysfunction. MiRNA-34a, miR-210, miR-690, and miR-467a-5p are related to insulin metabolism. Under the infiltration of adipose tissues, miR-34a in macrophages is up-regulated and inhibits Krüppel-like factor 4 (Klf4), which is able to inhibit the anti-inflammatory polarization of macrophages. miRNA is positively correlated with insulin resistance and metabolic inflammatory parameters (207). The macrophages in adipose tissues directly target the NADH dehydrogenase ubiquinone 1 α subcomplex 4 (NDUFA 4) gene by releasing miR-210 and promoting the onset of obesity-related diabetes by regulating glucose uptake and mitochondrial CIV activity (208). Anti-inflammatory M2-type macrophages are important to maintain normal metabolic homeostasis. M2-polarized bone marrow-derived macrophages (BMDM) secrete exosomes (Exos) containing miRNA, and miR-690 in the exosomes acts as an insulin sensitizer by inhibiting NAD kinase (NADK), thereby improving glucose tolerance (209). MiR-467a-5p, by targeting thrombospondin 1 (THBS1), increases the infiltration of macrophages in adipose tissues, increases the level of IL-6 in adipose tissues, and can prevent insulin resistance (210). Both miR-505-3p and miR-29 have pro-inflammatory effects. miR-505-3p is down-regulated and promotes the expression of the transcription factor (RUNX1). CCR3, CCR4, CXCR, and RUNX1 are increased in MΦ (macrophage), and this increase promotes pro-inflammatory macrophages (211). The miR-29 exosome promotes inflammation by promoting the recruitment and activation of circulating monocytes and macrophages in a TNF-receptor-associated factor 3 (TRAF3) dependent manner (212). Furthermore, our results show that pancreatic β cells regulate systemic inflammatory tone and glucose homeostasis through miR-29 in response to nutrient overload (212). Persistence of pro-inflammatory M1 macrophages in diabetic wounds contributes to the persistence of chronic inflammation in diabetic wounds. The persistence of M1 macrophage phenotype and its failure to remodel into M2-type macrophages play a key role in diabetic wound injury. The level of miR-21 has staged characteristics in diabetic wound healing. In the early and late stages of diabetic wound repair, miR-21 level is high, while in the middle stage of trauma, miR-21 level is significantly low. In macrophages, M1-polarized bacteriophage showed up-regulation of miR-21 and the pro-inflammatory factors IL-1β, TNF-α, and IL-6. In addition, hyperglycemia induces NOX2 expression and ROS production through the HG/miR-21/PI3K/NOX2/ROS signaling cascade. Dysregulation of miR-21 may lead to abnormal inflammation and persistent M1 macrophage polarization in diabetic wounds (225). The down-regulation of miR-712 reduces and inhibits the phosphorylation of p38 and ERK1/2 kinases and inhibits the pro-inflammatory transformation of macrophages by promoting the expression of apotant infectious gene LRRk2 (213). MiR-128-2 and miR-467a-5p participate in the regulation of macrophage cholesterol transport by inhibiting ATP-binding cassette transporter A1 (ABCA1), ATP-binding cassette subfamily G member 1 (ABCG1) and Retinoid X receptors α (RXRα) (24, 214).

##### 4.2.2.3 Rheumatoid Arthritis

Rheumatoid arthritis is chronic inflammatory arthritis that can lead to irreversible cartilage and bone damage, characterized by persistent synovitis, systemic inflammation, and autoantibodies (226). The down-regulation of miR-221-3p promotes the expression of Janus kinase 3 tyrosine-protein kinase (JAK3) and drives M2 macrophages to show M1 cytokine characteristics, resulting in weakened anti-inflammatory response and enhanced pro-inflammatory response (215). The upregulation of miR-29b targets the high-mobility group box-containing protein 1 (HBP1) and promotes the persistent existence of CD14-positive peripheral blood mononuclear cells (PBMs) at inflammatory sites (216). Up-regulation of miR-20a inhibits receptor activation of NF-κB ligand (RANKL), thereby inhibiting the proliferation and differentiation potential of osteoclasts (219). MiR-132 and miR-574-5p target COX2 and TLR 7/8 signaling, respectively, to promote osteoclastogenesis and intensify rheumatoid arthritis (217, 218). MiR-6089 inhibits lipopolysaccharide (LPS)-induced cell proliferation and activation of macrophase-like THP-1 cells by inhibiting the level of TLR4 (220).

##### 4.2.2.4 Infectious Bowel Disease

Infectious bowel disease (IBD) is a chronic and recurrent inflammatory bowel disease that is an abnormal immune response to intestinal microflora triggered by environmental factors in susceptible hosts. MiR-590-3p can activate the transcription regulated by YAP/β-catenin in macrophages, reduce inflammatory signals and promote epithelial regeneration by directly targeting large tumor suppressor, homolog 1 (LATS1) (222). The down-regulation of miR-148a increases the levels of GP130, inhibitor of nuclear factor kappa-B kinase α (IKKα), IKKβ, interleukin 1 receptor type 1 (IL1R1), and TNF receptor superfamily member 1b (TNFR2), resulting in the decreased activation of NF-κB and signal transducer and activator of transcription 3 (STAT3) in macrophages and colon tissues, and promotion of colitis (221). MiR-378a-5p, on the other hand, plays a vital role in the repair of colitis by targeting to nod-like receptor family, pyrin domain-containing 3 (NLRP3) (223).

##### 4.2.2.5 Other Chronic Diseases

In liver cirrhosis and idiopathic pulmonary fibrosis, miR-142-5p prolongs STAT6 phosphorylation by inhibiting suppressor of cytokine signaling 1 (SOCS1) protein, leading to increased IgE, eosinophil infiltration, fibroblast proliferation and collagen synthesis, and aggravates tissue fibrosis (26). MiR-130a-3p attenuates its repression of peroxisome proliferator-activated receptor γ (PPARγ), which coordinates STAT6 signaling, and also promotes tissue fibrosis (26). In systemic lupus erythematosus (SLE), downregulation of miR-4512 leads to high expression of TLR4 and CXCL2 in macrophages, which release more pro-inflammatory factors (23). In adult-onset still’s disease (AOSD), miR-20a is upregulated in macrophages and suppresses the expression of the proinflammatory factor IL-18 (149). PKCδ (protein kinase C δ) is an important mediator of inducing Mϕ polarization. In myocardial infarction, miR-181b regulates macrophage polarization by targeting PKCδ. We summarize the above content in **Table 2**.

## 5 Conclusion and Future Perspectives

In this paper, the latest progress of miRNA in regulating neutrophils and macrophages is reviewed. miRNAs can determine the resolution of inflammatory responses by regulating the functions of neutrophils and macrophages, and thus serve as potential therapeutic targets for chronic diseases. miRNA can regulate the level of inflammatory factors in injured or infected sites by influencing the differentiation and function of neutrophils and the formation of NET, play a role in polycytosis of self-apoptosis, and participate in the development of chronic diseases through oxidation and hydrolysis derived from phagocytosis and killing. For macrophages, miRNA mainly participates in chronic diseases by regulating the polarization, phagocytosis, efferent cytopenia, and lipid metabolism of macrophages, and repairing, regenerating, and fibrosis of tissues.

However, it is not difficult to find from the foregoing that most of the current effects of miRNAs are concentrated in a certain part of a specific disease. For example, miR-223-3p is anti-inflammatory in the context of sepsis (135) while pro-inflammatory in the context of COPD (147). We can conclude from this that the effects of miRNAs are environment-specific, that is, the functions of miRNAs on neutrophils and macrophages are highly dependent on surrounding environmental factors, which makes miRNAs have inevitable side effects. Therefore, extensive experiments are needed to evaluate the global regulatory network of miRNAs to determine the therapeutic utility of miRNAs before they can be put into the clinic.

## Author Contributions

PY and JZ: Conceptualization, Methodology, Funding acquisition. YW: Writing-Original draft preparation and Reviewing. XL: Visualization and Investigation. PX: Software and Supervision. ZL: Validation and Editing. XF and YS: Reviewing and Supervision. All authors contributed to the article and approved the submitted version.

## Funding

This research was supported by grants from the Key Science and Technology Research Project of Education Department of Jiangxi Province (GJJ180004).

## Conflict of Interest

The authors declare that the research was conducted in the absence of any commercial or financial relationships that could be construed as a potential conflict of interest.

## Publisher’s Note

All claims expressed in this article are solely those of the authors and do not necessarily represent those of their affiliated organizations, or those of the publisher, the editors and the reviewers. Any product that may be evaluated in this article, or claim that may be made by its manufacturer, is not guaranteed or endorsed by the publisher.
